# The Combined Value of Type2 Inflammatory Markers in Chronic Obstructive Pulmonary Disease

**DOI:** 10.3390/jcm11102791

**Published:** 2022-05-16

**Authors:** Yunhuan Liu, Guanhua Ma, Yan Mou, Xuanqi Liu, Wenjia Qiu, Yang Zheng, Huili Zhu, Haiyan Ge

**Affiliations:** Department of Respiratory and Critical Care Medicine, Huadong Hospital, Fudan University, Shanghai 200040, China; 20211280001@fudan.edu.cn (Y.L.); ma_guanhua@139.com (G.M.); mouyan321@163.com (Y.M.); 19211280001@fudan.edu.cn (X.L.); 18211280001@fudan.edu.cn (W.Q.); cbxzhsun@126.com (Y.Z.)

**Keywords:** COPD, exacerbation, fraction of exhaled nitric oxide, eosinophil, type2 immune response

## Abstract

The roles of type2 inflammatory markers in chronic airway diseases have been assessed in previous studies. However, the relationship between the combined value of these biomarkers and chronic obstructive pulmonary disease (COPD) has not been fully elucidated. We aimed to investigate the roles of the combined value of the fraction of exhaled nitric oxide (FeNO) level and blood eosinophil count in COPD and the predictive capability of these biomarkers. In total, 266 patients were included in our analysis. When the two type2 biomarkers were assessed separately, there were limited correlations between either increased FeNO level or blood eosinophil count and decreased incidence of total exacerbation or frequency of mild exacerbation. Combining these two biomarkers strengthened their association with both incidence and frequency of acute exacerbation. In addition, during further assessment, simultaneously increased FeNO level and blood eosinophil count were associated with both mild and moderate acute exacerbation. Among the subjects included in this analysis, although the predictive capability was improved when these two biomarkers were combined, the improvement was not statistically significant, indicating the need to increase the sample size. The combination of FeNO level and blood eosinophil count exhibited strong and independent additive value in the assessment of acute exacerbation in COPD; simultaneously increased FeNO level and blood eosinophil count played a protective role in progression of COPD.

## 1. Introduction

Chronic obstructive pulmonary disease (COPD) is one of the most common respiratory diseases in elderly people worldwide. The Global Burden of Disease Study 2017 reported that 3.2 million people died from COPD in 2017, which was 23% higher than that in 1990 [1]. Meanwhile, Wang et al. elucidated that an estimated 99.9 million people were currently influenced by this disease, and the prevalence of COPD was up to 13.7% in people over 40 years old [2]. During the time of COVID-19, COPD, no doubt, was a huge inconvenience for patients and an inestimable burden to society. Acute exacerbation of COPD has long been considered to be an important hallmark in the progression of COPD. Frequent exacerbations of COPD have been associated with an adverse outcome and deteriorated quality of life [3]. Previous studies have pointed out that acute exacerbation of COPD could be triggered by infections and environmental factors [4]. Currently, we still had an incomplete understanding of risk factors and the mechanism of acute exacerbation of COPD.

It has commonly been believed that the pathogenesis of COPD was associated with an increased number of macrophages, together with activated neutrophils and lymphocytes induced by cigarette smoking, which were characteristics of type1 inflammation, while type2 immune responses were shown in specific scenarios [5]. There is still controversy over the role of type2 immune response in COPD. According to some recent research, type2 inflammatory markers (such as fraction of exhaled nitric oxide (FeNO), sputum, blood eosinophil counts, and IgE) are also significant factors relating to progression and treatments of COPD [6]. Some research has even reported that the classification of chronic obstructive airway disease (COAD) according to underlying immunologic mechanism (Th2 high and Th2 low) was more effective in clinical practice than classical diagnostic labels (asthma, COPD, asthma-COPD overlap syndrome (ACOS), etc.) [5]. In recent years, many cohort studies have discussed the relationships between type2 inflammatory markers and COPD. Patients with different FeNO levels have shown differences in distribution of age, gender, and smoking status [7,8]. These type2 inflammatory markers have also been proven to be associated with lung functions of COPD [9]. Quality of life and prognosis have also been linked to these markers [10,11]. Moreover, some studies have elucidated the relationship between eosinophilia and immune response in the airway, for instance, immunoglobulin activity and B cell activity [12]. The sputum microbiome has also been shown to be influenced by blood eosinophil counts [13]. In addition, recently, several therapies targeting type2 inflammatory markers have been studied. The monoclonal antibodies targeting the IL-5 signaling pathway, for example, mepolizumab and benralizumab, exhibited elevating efficacy in reducing the rate of exacerbations in COPD patients with higher blood eosinophil counts [14,15]. In addition, triple therapy with inhaled corticosteroid/long-acting muscarinic antagonist/long-acting β_2_-Agonist (ICS/LAMA/LABA) and dual therapies with ICS/LAMA or ICS/LABA have shown different efficacies in COPD patients with different blood eosinophil count levels, suggesting the priority of these therapies should be considered with the blood eosinophil count of patients [16]. It has been shown in previous randomised controlled trials (RCTs) that the response to ICS differed among COPD patients having different levels of eosinophilic airway inflammation no matter whether asthma had been diagnosed or not [17]. These studies have clarified that, in a time of precise medicine, the heterogenicity of COPD, especially COPD with increased type2 inflammatory markers, needs to be taken into consideration in the treatment of this disease.

Nevertheless, the heterogenicity of COPD patients with increased type2 inflammatory makers has not been fully elucidated. Therefore, the influence of the combination of type2 inflammatory markers on acute exacerbation in COPD progression attracted our attention. Although FeNO level and blood eosinophil count are both related to type2 inflammation, their underlying mechanisms differ from each other. Increased FeNO level has been demonstrated to locally activate the IL-4 and IL-13 pathways, while increased eosinophil count was related to IL-5, which was not influenced by IL-4 and IL-13 [18,19]. These differences formed the basis of this combination. To further illustrate the heterogenicity of COPD and guide clinical practice, we sought to identify the role of the combined value of FeNO level and blood eosinophil count in COPD phenotype and acute exacerbation of COPD using data from the Shanghai COPD Investigation on Comorbidity Program (SCICP, ChiCTR2000030911). In this retrospective study, we investigated the relationship between the combination of these type2 inflammatory markers and acute exacerbation of COPD through an analysis of patients’ results from clinical examinations and their prognosis in the previous year. Whether these markers have predictive capability in the progression of COPD separately or combined was also analyzed in this study.

## 2. Materials and Methods

### 2.1. Study Design and Participants

**Figure 1:**
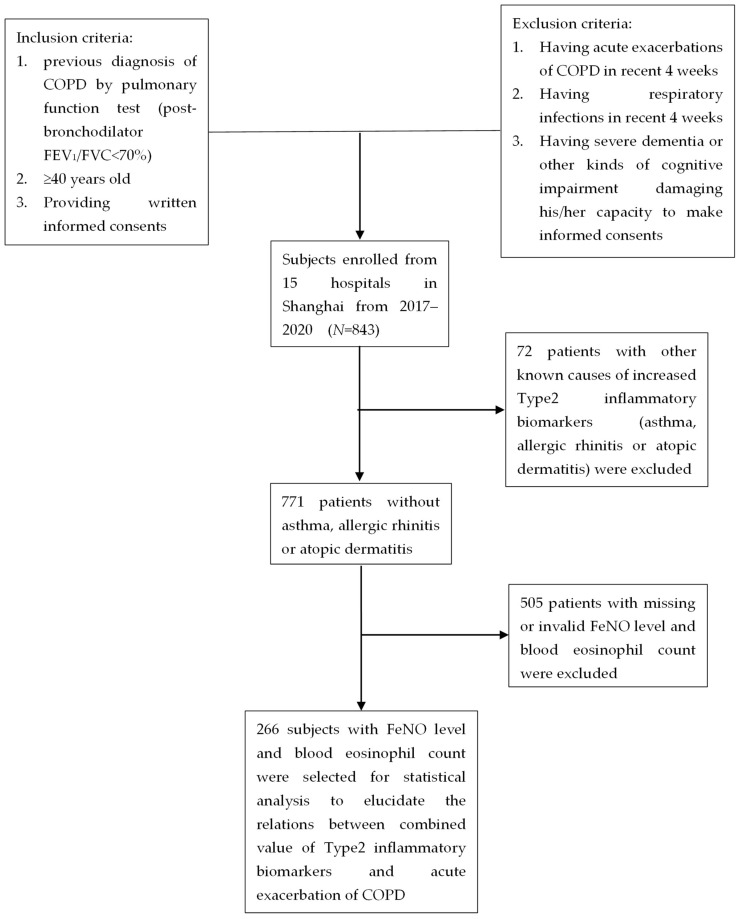
Consort diagram of the study.

There were 266 patients included in our analysis from the Shanghai COPD Investigation on Comorbidity Program (SCICP, ChiCTR2000030911). Each patient in this program completed a comprehensive questionnaire, underwent a thorough physical examination, and provided blood for the biochemical analyses. The pulmonary function tests were done by professional medical technicians and repeated twice to obtain the best results. The exacerbation of COPD was assessed by professional physicians and was recorded. The patients were followed up regularly every 3 months. The exacerbation information was collected through inquiring and looking up medical records. The inclusion criteria were the following: (1) a previous diagnosis of COPD, which was defined as dyspnea, chronic cough, and/or sputum production along with post-bronchodilator (BD) forced expiratory volume in 1 s/forced vital capacity (FEV_1_/FVC) ratio < 70%, according to the Global Initiative for Chronic Obstructive Lung disease (GOLD); (2) age ≥ 40 years; (3) provision of written informed consent. The exclusion criteria included: (1) having acute exacerbations of COPD in the 4 weeks before the enrollment; (2) having respiratory infections in the 4 weeks before the blood collection; (3) having severe dementia or other kinds of cognitive impairment damaging the subject’s capacity to make informed consent. In order to assess the relationship between type2 inflammatory markers and exacerbation, 72 subjects with a history of asthma, allergic rhinitis, or atopic dermatitis, which could influence the type2 inflammatory markers, were not included in our study. There were 505 subjects with missing or invalid FeNO level or blood eosinophil count information who were excluded. Finally, 266 subjects with FeNO levels and blood eosinophil counts were included in this study. (Figure 1). The related information of these patients including their medical records and results of laboratory exams were used for analysis. The present study was approved by the Ethical Committee of the Huadong Hospital.

### 2.2. FeNO Measurement

The FeNO level was measured by applying a Sunvou device (Sunvou Medical Electronics Co., Ltd., Wuxi, China), in accordance with the recommendations of European Respiratory Society (ERS) and American Thoracic Society (ATS) [20]. The patient was seated comfortably, with an inserted mouthpiece, and asked to exhale at a flow rate of 0.05 L/s after inhaling through the mouth to total lung capacity (TLC), or near TLC if it was difficult for the patient to inhale to TLC. The use of a nose clip was discouraged to avoid contamination of the sample with nasal NO. During the test, breath-holding was also prohibited to avoid its influence on FeNO level. In addition, spirometry was performed after the FeNO analysis. Patient refrained from eating to prohibit the influence of nitrate or nitrate-containing foods, such as lettuce, on the results of the tests. Smoking and alcohol could affect the results of the assessment. Therefore, patients were asked not to smoke or drink alcohol one day before the test when physicians prescribed this test to them. Moreover, strenuous exercise was avoided before the assessment, because of the results of previous research [21]. The FeNO level was expressed in parts per billion (ppb). The median of the FeNO level in this study was 35 ppb. Some previous research has demonstrated that a high FeNO level (FeNO level > 50 ppb and > 35 ppb in children) could be used to indicate eosinophilic inflammation. COPD with mixed inflammatory phenotype was included in this category [22]. Therefore, the following analysis was based on a cutoff value of 35 ppb.

### 2.3. Blood Eosinophil Count

White blood cell counts were measured on peripheral blood samples using an ADVIA Hematology System (Siemens Healthcare, Munich, Germany). The blood eosinophil counts were presented in ×10^9^/L, along with other leukocyte subpopulations. A blood eosinophil count < 0.3 × 10^9^/L was considered to be normal, while a blood eosinophil count ≥ 0.3 × 10^9^/L was considered to be increased. It has been demonstrated in previous studies that this cutoff value was related to disease severity and incidence of acute exacerbation in COPD [23].

### 2.4. Pulmonary Function Test

The flow-volume curves were obtained with a Jaeger Toennies spirometer (Höchberg, Germany), according to the American Thoracic Society and European Respiratory Society (ATS/ERS) guidelines [24,25]. The procedure and key points of this test were explained to the patients before the actual test. Spirometry was performed by trained technicians. The parameters including FEV1 post BD, FVC post BD, FEV1/FVC post BD, and FEV1/predict post BD were used in our analysis.

### 2.5. Definition of Acute Exacerbation

COPD patients were confirmed using spirometry in this cohort with the presence of FEV_1_/FVC < 0.7 in post-bronchodilator spirometry testing in accordance with GOLD 2018 [26]. Acute exacerbation of COPD in this cohort was defined as acute worsening of respiratory symptoms, such as cough, dyspnea, and expectoration, leading to additional treatment. Acute exacerbation of COPD was divided into mild, moderate, and severe acute exacerbation. Mild acute exacerbation could be controlled with single short-acting bronchodilators treatment. Moderate acute exacerbation could be relieved by short-acting bronchodilators and antibiotics, with or without oral corticosteroid. Severe acute exacerbation referred to exacerbation requiring emergency admission or ICU transferring [27]. The exacerbation of COPD and its levels were identified in two ways, i.e., from reading the medical record and from inquisition during the patients’ visits.

### 2.6. Other Information

Body mass index (BMI) was defined as the body mass divided by the square of the body height, and was presented in units of kg/m^2^. The classification of BMI was based on Chinese standards [28]. The major adult BMI classifications were underweight (under 18.5 kg/m^2^), normal weight (18.5 to 23.9 kg/m^2^), overweight (24 to 27.9 kg/m^2^), and obese (over 28 kg/m^2^). The smoking status was classified by pack-years. A heavy smoker was defined if a subject’s pack-year was over 15 pack-years [29]. The modified Medical Research Council Scale (mMRC) and St. George’s Respiratory Questionnaire (SGRQ) were used to evaluate the symptoms and quality of life of patients, respectively. These two evaluation were done by professional physicians. A 6-minute walk test (6 MWT) was performed with patients using usual oxygen flows and walkers if needed. The walking distances were recorded at the end of the 6 MWT. The oxygen-pulsed saturation (SpO_2_) and heart rate (HR, beats/min) were monitored during the tests. Information on asthma, allergic rhinitis, and atopic dermatitis was self-reported.

### 2.7. Statistical Analyses

Statistical analyses were performed using STATA/SE 16.0 for Windows (StataCorp, College Station, TX, USA). Continuous variates were presented as mean ± standard deviation or median (25th and 75th percentile), while categorical variates were presented as n or *n* (%). First, the differences between the characteristics of groups with different FeNO levels and blood eosinophil counts were analyzed using the Kruskal–Wallis test. An adjusted *p*-value was used for multiple comparisons among groups. Second, logistic regression and Poisson regression were used to elucidate the relationship between exacerbation and increased FeNO level and blood eosinophil count. Third, receiver operating characteristic (ROC) curves were utilized to evaluate the predictive capability of FeNO level and blood eosinophil count. Smoking status was adjusted in the univariate analyses and ROC analyses. All analyses for the two biomarkers were performed separately and combined.

## 3. Results

### 3.1. Subject Characteristics and Classifications

There were 266 patients from the Shanghai COPD Investigation on Comorbidity Program (SCICP, ChiCTR2000030911) included in our study to analyze the correlation between type2 inflammatory markers (such as FeNO level and blood eosinophil count) and the occurrence of acute exacerbation in COPD. Figure 1 presents the consort diagram of the study. The subjects included in our analysis were predominantly male (87.59%) with a median age of 72 (65.50–80.00) years. Among the 266 individuals, 66.54% of the subjects were former or current smokers with significant smoking exposure. The median pack-year was 25 (9–47.5) pack-years. Most of the subjects had mild to severe airflow limitation; the median post-bronchodilator FEV_1_/predict was 59.30% (43.30–70.10%). The median score on the St George’s Respiratory Questionnaire (SGRQ) was 37 (32–59), while the median 6-minute walking distance (m) was 320 (300–350), indicating that the majority of the subjects in this study suffered from low quality of life and deteriorated motor capacity. Among the 266 subjects enrolled in this analysis, 52.85% had at least 1 exacerbation in the previous year. The mean exacerbation frequency was 1.29 in this study. The median value of the FeNO level (ppb) was 35 (23–50), while the median value of the eosinophil count (×10^9^/L) was 0.21 (0.10–0.40) (Table 1).

The subjects included in our analysis were divided into three groups based on their FeNO levels and blood eosinophil counts (Table 2): Group A, subjects with normal FeNO levels and blood eosinophil counts (*n* = 120); Group B, subjects with increased FeNO levels or blood eosinophil counts (*n* = 90); Group C, subjects with increased FeNO levels and blood eosinophil counts (*n* = 56). An older age, lower pack-years, higher post-bronchodilator FEV_1_/FVC and post-bronchodilator FEV_1_/predict, lower post-bronchodilator FVC, and more limited motor capacity were all associated with an increased possibility of being in Group C (Table 2). In addition, lower incidence and total frequency of acute exacerbation in one year were both associated with Group C (Table 2 and Supplementary Figure S1). There were no significant differences in gender, BMI, smoking history, post-bronchodilator FEV_1_, and St George’s Respiratory Questionnaire (SGRQ) score among the 3 groups.

A weak-to-moderate correlation was found between FeNO levels and blood eosinophil counts (r = 0.35, *p* < 0.001) (Supplementary Figure S2).

### 3.2. Increased FeNO Level in Relation to Incidence and Frequency of Acute Exacerbation

**Figure 2:**
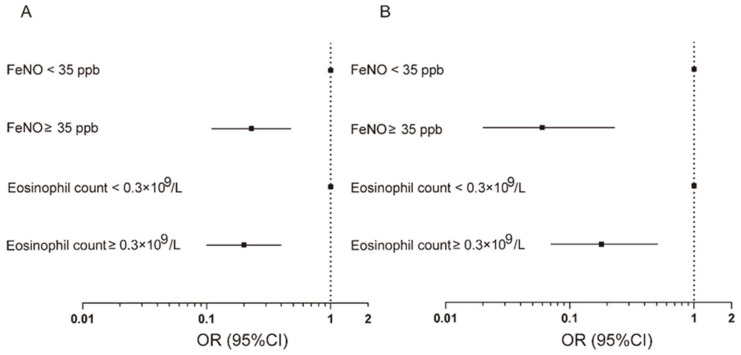
Separate association of increased FeNO level and blood eosinophil count with incidence of acute exacerbation of COPD: (**A**) Smoking status adjusted; (**B**) multivariable adjusted. Logistic regression models were used. Multivariable adjustment included age, gender, BMI, smoking status, FEV_1_ post-BD% pred, SGRQ, 6-minute walking distance. FeNO, exhaled nitric oxide fraction; BMI, body mass index; FEV_1_, forced expiratory volume in 1 s; BD, bronchodilator; SGRQ, St. George’s Respiratory questionnaire.

**Figure 3:**
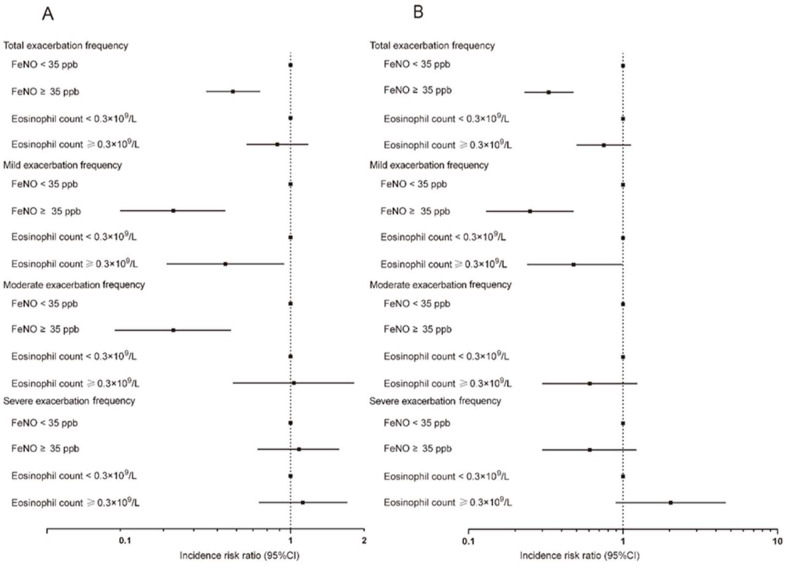
Separate association of increased FeNO level and blood eosinophil count with frequency of various degrees of acute exacerbation of COPD: (**A**) Smoking status adjusted; (**B**) multivariable adjusted. Poisson regression models were used. Multivariable adjustment included age, gender, BMI, smoking status, FEV_1_ post-BD% pred, SGRQ, and 6-minute walking distance. FeNO, exhaled nitric oxide fraction; BMI, body mass index; FEV_1_, forced expiratory volume in 1 s; BD, bronchodilator; SGRQ, St. George’s Respiratory Questionnaire.

When these two type2 inflammatory biomarkers were analyzed separately, an increased FeNO level was associated with reduced risk of various degrees of acute exacerbation from the aspect of both incidence and frequency. As compared with subjects with FeNO levels < 35 ppb, the smoking status-adjusted odds ratio (95% CI) for acute exacerbation was 0.23 (0.11–0.48) for subjects with FeNO levels ≥ 35 ppb (Supplementary Table S1 and Figure 2). Meanwhile, as compared with subjects with FeNO levels < 35 ppb, the smoking status-adjusted incidence rate ratio for total acute exacerbation was 0.58 (0.45–0.75) for subjects with FeNO levels ≥ 35 ppb (Supplementary Table S2 and Figure 3). The corresponding incidence rate ratios were 0.33 (0.20–0.54) and 0.33 (0.19–0.57) for mild and moderate acute exacerbation. Adjustment for additional potential confounders gave similar results (Supplementary Table S3 and Figure 3). The multivariable-adjusted odds ratio (95% CI) for acute exacerbation was 0.06 (0.02–023) for subjects with FeNO levels ≥ 35 ppb as compared with subjects with FeNO levels < 35 ppb (Supplementary Table S1 and Figure 2). In addition, the multivariable-adjusted incidence rate ratio for total acute exacerbation was 0.33 (0.23–0.48) for subjects with FeNO levels ≥ 35 ppb (Supplementary Table S2 and Figure 3). The corresponding incidence rate ratio was 0.25 (0.13–0.48) for mild exacerbation after additional adjustment for potential confounders (Supplementary Table S3 and Figure 3).

### 3.3. Increased Blood Eosinophil Count in Relation to Incidence and Frequency of Acute Exacerbation

It was demonstrated in the logistic regression analysis and Poisson regression analysis that an increased blood eosinophil count was associated with reduced risk of acute exacerbation of COPD. As compared with subjects with blood eosinophil count < 0.3 × 10^9^/L, the smoking status-adjusted odds ratio (95% CI) for acute exacerbation was 0.20 (0.10–0.40) for subjects with blood eosinophil count ≥ 0.3 × 10^9^/L. Otherwise, when additional potential confounders were adjusted, the corresponding odds ratio for subjects with blood eosinophil count ≥ 0.3 × 10^9^/L was 0.18 (0.07–0.51) (Supplementary Table S1 and Figure 2). For frequency of total acute exacerbation, the smoking status-adjusted incidence rate ratio was 0.88 (0.66–1.18) for subjects with blood eosinophil count ≥ 0.3 × 10^9^/L as compared with subjects with blood eosinophil count < 0.3 × 10^9^/L, and the result remained similar after adjustment of additional potential confounders (Supplementary Table S2 and Figure 3). As compared with subjects with blood eosinophil count < 0.3 × 10^9^/L, the incidence rate ratios were 0.54 (0.31–0.94) and 0.48 (0.24–0.99) for mild exacerbation when smoking status and additional potential confounders were adjusted, respectively. The results were attenuated for moderate and severe exacerbation after both adjustments for smoking status and additional potential confounders (Supplementary Table S3 and Figure 3).

### 3.4. Simultaneously Increased FeNO Level and Blood Eosinophil Count in Relation to Acute Exacerbation and Their Predictive Values

**Figure 4:**
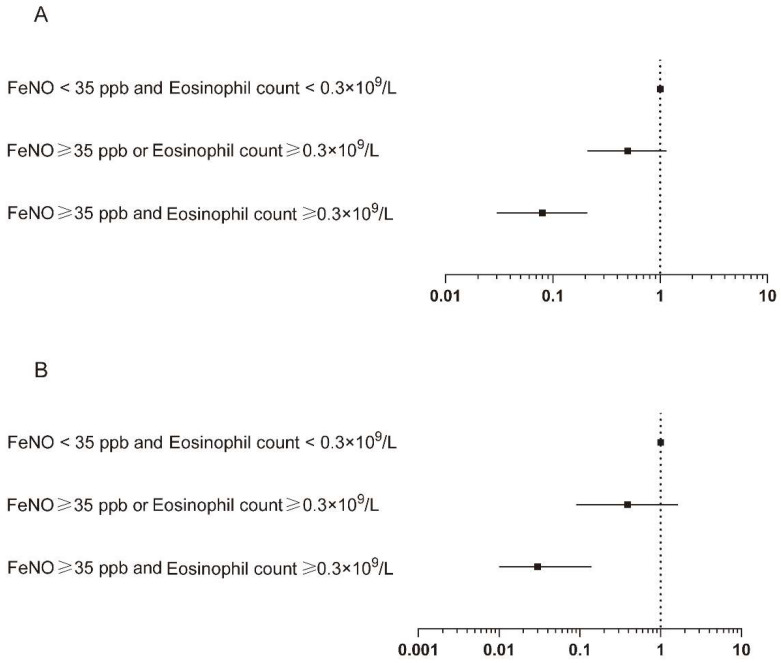
Combined association of increased FeNO level and blood eosinophil count with incidence of acute exacerbation of COPD: (**A**) Smoking status adjusted; (**B**) multivariable adjusted. Logistic regression models were used. Multivariable adjustment included age, gender, BMI, smoking status, FEV_1_ post-BD% pred, SGRQ, and 6-minute walking distance. FeNO, exhaled nitric oxide fraction; BMI, body mass index; FEV_1_, forced expiratory volume in 1 s; BD, bronchodilator; SGRQ, St. George’s Respiratory Questionnaire.

**Figure 5:**
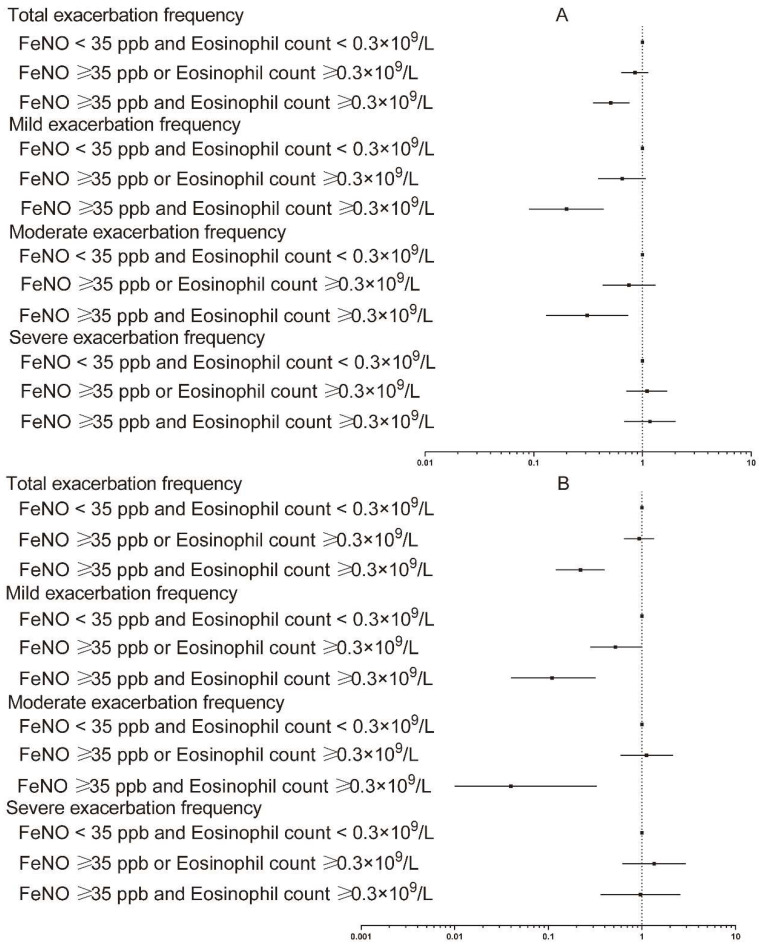
Combined association of increased FeNO level and blood eosinophil count with frequency of various degrees of acute exacerbation of COPD: (**A**) Smoking status adjusted; (**B**) multivariable adjusted. Poisson regression models were used. Multivariable adjustment included age, gender, BMI, smoking status, FEV_1_ post-BD% pred, SGRQ, and 6-minute walking distance. FeNO, exhaled nitric oxide fraction; BMI, body mass index; FEV_1_, forced expiratory volume in 1 s; BD, bronchodilator; SGRQ, St. George’s Respiratory Questionnaire.

When the two type2 inflammatory biomarkers were analyzed simultaneously, increased FeNO level and blood eosinophil count was associated with reduced risk of mild and moderate exacerbation. As compared with subjects in Group A, the smoking status-adjusted odds ratios for acute exacerbation were 0.50 (0.21–1.15) for subjects in Group B, and 0.08 (0.03–0.21) for subjects in Group C. The results were similar after additional adjustment for potential confounders. The multivariable-adjusted odds ratios were 0.39 (0.09–1.64) and 0.03 (0.01–0.14) for subjects in Group B and Group C as compared with subjects in Group A, respectively (Supplementary Table S4 and Figure 4). It was demonstrated in the Poisson regression analysis that the smoking status-adjusted incidence rate ratio for total exacerbation was 0.85 (0.64–1.13) for subjects in Group B, while the corresponding incidence rate ratio was 0.51 (0.35–0.76) for subjects in Group C as compared with subjects in Group A. Adjustment for additional potential confounders gave similar results (Supplementary Table S5 and Figure 5). As compared with subjects in Group A, subjects in Group C tended to have lower risk of exacerbation, especially the risk of mild or moderate exacerbation. The smoking status-adjusted incidence rate ratios were 0.20 (0.09–0.44) and 0.31 (0.13–0.74) for subjects in Group C for mild and moderate exacerbation, respectively. The results were attenuated for subjects in Group B. When additional potential confounders were adjusted, the incidence rate ratios were 0.11 (0.04–0.32) and 0.04 (0.01–0.33) for mild and moderate exacerbation, respectively, for subjects in Group C as compared with subjects in Group A. The corresponding incidence rate ratio for subjects with increased FeNO levels or blood eosinophil counts was statistically significant only in mild exacerbation. There were no clear associations observed in moderate and severe exacerbation (Supplementary Table S6 and Figure 5).

Among individuals in our analysis, no difference was observed between the predictive capability of FeNO level and blood eosinophil count. Although their predictive capability was improved when these two biomarkers were combined, it was not statistically significant, indicating the necessity of increasing the sample size. The AUC (95% CI) value for predicting acute exacerbation in one year was 0.76 (0.69–0.83) for FeNO level ≥ 35 ppb, while the AUC (95% CI) value for blood eosinophil count ≥ 0.3 × 10^9^/L was 0.76 (0.69–0.83). The AUC (95% CI) value for FeNO level ≥ 35 ppb and blood eosinophil count ≥ 0.3 × 10^9^/L combined was 0.79 (0.72–0.86) (Supplementary Table S7 and Supplementary Figure S3).

## 4. Discussion

COPD is a chronic respiratory disease associated with high morbidity and mortality rate. Acute exacerbation of COPD, which is mostly caused by infectious and environmental factors, is one of the most important events in COPD progression. Acute exacerbation of COPD has been associated with increased mortality in several cohort studies [30,31]; 25% of patients in acute exacerbation required ICU admission, which further increased the economic burden of COPD [32]. In addition, frequent exacerbation would severely worsen patients’ quality of life. Therefore, facile and effective biomarkers which have the capability of assessing the risk of exacerbation are urgently needed in clinical practice. The correlation between some facile clinical markers and acute exacerbation of COPD has long attracted the attention of many researchers who have aimed to build a precise predictive grading system for screening patients with high risk of exacerbation. FeNO level and blood eosinophil count, which are significant type2 inflammatory biomarkers, have been analyzed in several previous clinical studies concerning COPD and acute exacerbation of COPD because of their accessibility and convenience [9,33,34,35]. However, the results of these studies have been inconsistent and further investigation of these two biomarkers, especially the combination of these two biomarkers, is required. In this retrospective cohort study, our team elucidated that subjects with increased FeNO levels and subjects with increased blood eosinophil counts both tended to have reduced risk of acute exacerbation, especially mild excerbation, as compared with subjects with normal FeNO levels and normal blood eosinophil counts. This inclination could be seen when these two biomarkers were analyzed separately, and the results were strengthened when these two biomarkers were combined. When the combination of these two type2 inflammatory biomarkers was analyzed, an increased FeNO level and blood eosinophil count was associated with reduced incidence and frequency of exacerbation, especially mild and moderate exacerbation. Simultaneously increased FeNO level and blood eosinophil count also exhibited an association with relatively milder pulmonary function deterioration. The predictive capabilities were also assessed in our study and we found that the predictive capability of these two biomarkers did not differ from each other. Although their predictive capability was improved when the two biomarkers were combined, it was not statistically significant, indicating the necessity of increasing the sample size. Through this analysis, we intended to investigate the role of FeNO level and blood eosinophil count, especially the combination of these two biomarkers, in COPD. The relationship between these two biomarkers and acute exacerbation might provide a thorough understanding of COPD heterogenicity and precise guidance to clinical practice.

Exhaled nitric oxide (eNO), which was first detected in 1993, was used to evaluate airway responsiveness in the 1990s. Although it has long been considered to be a type2 inflammatory marker used in asthma assessment [36,37,38], recent research has also demonstrated that it was associated with asthma-COPD overlap (ACO) and COPD. It has been reported that FeNO level could be related to pulmonary function and symptom severity of ACO patients in meta-analysis and cohort studies [35,39,40]. Some research has illustrated that FeNO and other type2 inflammatory markers, for example, eosinophil count, could be used to differentiate between ACO and COPD [41]. A positive correlation between age and FeNO level was demonstrated in a previous research [7]. Similar age distribution was noticed in our analysis. In addition, active smoking and tobacco exposure were proven to decreased FeNO level in non-atopic individuals [42]. In our study, subjects with increased FeNO levels and blood eosinophil counts tended to have lower pack-years, which seemed to compensate the previous findings. Previous studies have reported that healthy males tended to have higher FeNO levels than females [43]. However, this gender distribution was not found in our study. A possible explanation could be that the subjects included in our analysis were predominantly male. In addition, 66.54% of the subjects were former or current smokers, and FeNO levels could be influenced by their smoking history.

Sputum or blood eosinophil counts, as type2 inflammatory biomarkers had long been considered associated with asthma and ACO. In COPD management, airway eosinophilic inflammation could improve the response to conventional treatment of COPD, for example budesonide [44,45]. Many previous studies focused on the sputum or blood eosinophil count during exacerbation [46]. However, in COPD exacerbation, many confounders could influence eosinophil counts both in sputum and blood stream. Our study concentrated on the role of blood eosinophil count in stable COPD, which could better reflect its predictive capability of acute exacerbation of COPD.

Our study presented that increased FeNO level and blood eosinophil count could be related to reduced incidence and frequency of various degrees of acute exacerbation of COPD. However, the role of the combination of FeNO level and blood eosinophil count has been controversial, and the results of various studies have been inconsistent. It was reported by a cohort analysis that increased blood eosinophil count was associated with a reduced mortality rate in COPD patients [33]. A retrospective study demonstrated that blood eosinophil count could predict a positive relationship with pulmonary function parameters [47]. A three-year prospective clinical trial also demonstrated that FeNO level elevation was linked to decreased exacerbation frequency [48]. In addition, in one of our team’s cohort analysis of the comorbidities of COPD, allergic rhinitis was found to play a protective role in acute exacerbation of COPD [49]. In concordance with these previous studies, our study demonstrated that increased FeNO level and blood eosinophil count could decrease the risk of acute exacerbation separately and combined. However, some cohort studies have reported that the correlations between these two biomarkers combined and acute exacerbation were not statistically significant [35]. In addition, a recent study reported that combining FeNO and blood eosinophil count could enhance the detection of future exacerbation of COPD [50]. There are several causes of these discrepancies. The blood eosinophil count and FeNO level could be influenced by the status of COPD. The blood eosinophil count and FeNO level in our research was taken when the patient was stable. These values could be affected if the patient was in the exacerbation period or if the patient’s COPD status could not be identified [51]. Second, the definition of exacerbation could influence the assessment of the relationship between blood eosinophil count and acute exacerbation in COPD. Mild exacerbation was included in the assessment of our research. Only moderate and severe exacerbation were included in some previous researches [52]. Overall, these results exhibit that the role of FeNO level and blood eosinophil count in COPD and the underlying mechanism of these two biomarkers in acute exacerbation require further investigation before they can be applied in clinical practice.

It has been demonstrated in previous studies that some patients with increased FeNO levels did not have an asthma history [53]. In addition, it was pointed out by one research by Annangi et al. that eosinophilic COPD could be independent of asthma history [54]. In our study, we focused on COPD patients with increased FeNO levels and blood eosinophil counts without asthma history. An investigation of the clinical attributes and the tendency of exacerbation of this definite group could provide a new perspective of COPD heterogenicity and guide precise therapeutic interventions for this group of patients.

## 5. Limitations

There were several limitation associated with this study that should be mentioned. The follow-up could be longer, and therefore, we could assess the persistence of the relations between type2 biomarkers and exacerbation of COPD. Our team is still undergoing a long-term follow-up of the cohort. In addition, the sample size might need to be enlarged to provide a more precise analysis of type2 immune response in COPD patients. Additionally, some other type2 inflammatory biomarkers (for example, IgE) need to be included in the analysis of type2 biomarkers in COPD. There might be some other causes of increased type2 inflammatory biomarkers, which should have been excluded in our following study. In addition, subjects with asthma were identified using the question, “Has a doctor or healthcare professional ever told that you have asthma?” Although, in previous studies the answer to this question was the most frequently used definition to define asthma history, we checked the patients’ medical records for confirmation and found that reliance on this question as definitive confirmation might lead to an underestimation of the prevalence of clinically confirmed asthma [55]. Meanwhile, the underlying mechanisms of the protective roles of these two biomarkers requires further elucidation.

## 6. Conclusions

The combination of FeNO level and blood eosinophil count exhibited strong and independent additive value in an assessment of the occurrence of acute exacerbation in COPD. Simultaneously increased FeNO level and blood eosinophil count played a protective role in the progression of COPD and decreased the incidence and frequency of acute exacerbation. The results of this research indicate that combining these two biomarkers might provide a thorough understanding of COPD heterogenicity and precise guidance for clinical decisions.

## Figures and Tables

**Table 1:** Baseline characteristics of the 266 subjects.

Characteristics	COPD
Age (years)	72 (65.50–80.00)
Males/females	233 (87.59)/33 (12.41)
BMI	23.50 (21.26–24.91)
**Smoking status**	
Never/former/current smoker	89 (33.46)/148 (55.64)/29 (10.90)
**Pack-years**	
Never smoker	NA
Former/current smoker	25 (9–47.5)
**Pulmonary function**	
FEV_1_ post BD (L)	1.24 (1.09–1.35)
FVC post BD (L)	1.96 (1.79–2.34)
FEV_1_/FVC post BD (%)	63.25 (50.83–66.52)
FEV_1_/predict post BD (%)	59.30 (43.30–70.10)
**mMRC**	
Grade 0	21 (7.89)
Grade 1	121 (45.49)
Grade 2	74 (27.82)
Grade 3	45 (16.92)
Grade 4	5 (1.88)
SGRQ	37 (32–59)
6-min walking distance (m)	320 (300–350)
**Exacerbations in previous year**	
Incidence	139 (52.85)
Total frequency of exacerbation	1.29 ± 2.24
FeNO (ppb)	35 (23–50)
Eosinophil count × 10^9^/L	0.21 (0.10–0.40)

Data are presented as mean ± SD, median (25th and 75th percentile), or *n* (%). COPD, chronic obstructive pulmonary disease; BMI, body mass index; FEV_1_, forced expiratory volume in 1 s; BD, bronchodilator; FVC, forced vital capacity; mMRC, Modified Medical Research Council Dyspnea Scale; SGRQ, St. George’s Respiratory Questionnaire; FeNO, exhaled nitric oxide fraction; NA, not applicable.

**Table 2:** Characteristics and classification of subjects.

	Groups		
Groups	A	B	C
Characteristics	Eosinophil count < 0.3 × 10^9^/L and FeNO < 35 ppb	Eosinophil count ≥ 0.3 × 10^9^/L or FeNO ≥ 35 ppb	Eosinophil count ≥ 0.3 × 10^9^/L and FeNO ≥ 35 ppb
*N*	120	90	56
Age (years)	69 (63–78)	72 (65–81)	76 (72–82)
Gender (males)	106 (88.33)	76 (84.44)	51 (91.07)
BMI	22.85 (20.58–25.70)	23.51 (21.74–24.97)	24.22 (22.12–24.97)
Underweight	12 (10.00)	7 (7.78)	2 (3.57)
Normal weight	67 (55.83)	44 (48.89)	22 (39.29)
Overweight	32 (26.67)	30 (33.33)	25 (44.64)
Obese	9 (7.50)	9 (10.00)	7 (12.50)
Smoking status			
Former and current smoker	81 (67.5)	64 (71.11)	32 (57.14)
Pack-years	40 (20–60)	35 (15–50)	9 (2.75–30)
≥15 pack-years	56 (46.67)	50 (55.56)	13 (23.21)
Lung function			
FEV_1_ post-BD (L)	1.20 (0.80–1.67)	1.22 (1.05–1.37)	1.25 (1.24–1.3)
FVC post-BD (L)	2.25 (1.89–2.98)	1.98 (1.88–2.48)	1.96 (1.91–1.99)
FEV_1_/FVC post-BD (%)	55.70 (42.20–65.31)	62.12 (46.61–66.85)	63.30 (63.20–63.85)
FEV_1_ post-BD% pred (%)	48.90 (37.70–60.00)	50.35 (39.90–67.78)	67.66 (61.40–72.37)
Quality of Life			
SGRQ	39 (27–52)	37 (32–47)	35 (32–37)
Motor capacity			
6-minute walking distance (m)	378 (276–420)	340 (310–373)	320 (310–330)
Previous exacerbations			
Incidence	67 (56.30)	61 (68.54)	11 (20.00)
Total frequency	1.69 ± 1.33	1.39 ± 1.86	0.69 ± 1.94

Data are presented as mean ± SD, median (25th and 75th percentile), or *n* (%). Results in boldface indicate a *p*-value less than 0.05. COPD, chronic obstructive pulmonary disease; BMI, body mass index; FEV_1_, forced expiratory volume in 1 s; BD, bronchodilator; FVC, forced vital capacity; SGRQ, St. George’s Respiratory Questionnaire; FeNO, exhaled nitric oxide fraction; NA, not applicable.

## Supplementary Materials

The following supporting information can be downloaded at https://www.mdpi.com/article/10.3390/jcm11102791/s1, Figure S1: Combination of normal or increased FeNO levels and blood eosinophil counts in relation to frequency of total acute exacerbation of COPD; Figure S2: Correlation between FeNO levels and blood eosinophil counts (r = 0.35, *p* < 0.001); Figure S3: Receiver operating characteristic curves (ROC) analysis for increased FeNO level and blood eosinophil count separately and combined; Table S1: Adjusted* odds ratios (95% CI) for incidence of acute exacerbation in 12 months if having singly increased FeNO levels (≥35 ppb) compared with having normal FeNO levels or having singly increased blood eosinophil counts (≥0.3 × 10^9^/L) compared with having normal blood eosinophil counts; Table S2: Adjusted* incidence-rate ratios for acute exacerbation in 12 months if having singly increased FeNO levels (≥35 ppb) compared with having normal FeNO levels and having singly increased blood eosinophil count (≥0.3 × 10^9^/L) compared with having normal blood eosinophil count; Table S3: Adjusted* incidence-rate ratios for different levels of acute exacerbation in 12 months if having singly increased FeNO levels (≥35 ppb) compared with having normal FeNO levels and having singly increased blood eosinophil count (≥0.3 × 10^9^/L) compared with having normal blood eosinophil count; Table S4: Adjusted* odds ratios (95% CI) for incidence of acute exacerbation in 12 months if having singly increased FeNO levels (≥35 ppb) or blood eosinophil counts (≥0.3 × 10^9^/L) or simultaneously increased FeNO levels and blood eosinophil counts compared with having both normal FeNO levels and blood eosinophil counts; Table S5: Adjusted* incidence-rate ratios (95% CI) for acute exacerbation in 12 months if having singly increased FeNO levels (≥35 ppb) or blood eosinophil counts (≥0.3 × 10^9^/L) or simultaneously increased FeNO levels and blood eosinophil counts compared with having both normal FeNO levels and blood eosinophil counts; Table S6: Adjusted* incidence-rate ratios (95% CI) for acute exacerbation in 12 months if having singly increased FeNO levels (≥35 ppb) or blood eosinophil counts (≥0.3 × 10^9^/L) or simultaneously increased FeNO levels and blood eosinophil counts compared with having both normal FeNO levels and blood eosinophil counts; Table S7: Predictive capabilities of increased FeNO level and blood eosinophil count.
