# Supplementary material for: The Combined Value of Type2 Inflammatory Markers in Chronic Obstructive Pulmonary Disease
Source: J Clin Med. 2022 May 16;11(10):2791. doi: 10.3390/jcm11102791 (PMC9144416; doi:10.3390/jcm11102791)
Supplement: Supplementary file 1 [file jcm-11-02791-s001.zip › jcm-1661430-supplementary.pdf]

Supplementary material

Supplementary Table S1. Adjusted * odds ratios (95% CI) for incidence of acute exacerbation in 12 months if having singly increased FeNO levels (/\\35ppb) compared with having normal FeNO levels or having singly increased blood eosinophil counts (/\\0.3 × 10^9/L) compared with having normal blood eosinophil counts. Multivariable Logistic Regression Model was used.

		Incidence of exacerbation in 12 months	
FeNO (ppb)	Eosinophil count (/L)	Smoking status -adjusted	Multivariable adjusted
/\\35		0.23 (0.11-0.48)	0.06 (0.02-0.23)
	/\\0.3 × 10^9	0.20 (0.10-0.40)	0.18 (0.07-0.51)

Results in boldface indicate a P value less than 0.05.

* Multivariable adjustment included age, gender, BMI, smoking status, FEV_1 post-BD% pred, SGRQ, 6 minutes walking distance.

FeNO: exhaled nitric oxide fraction; BMI: body mass index; FEV_1: forced expiratory volume in 1 s; BD: bronchodilator; SGRQ: St. George's Respiratory questionnaire.

Supplementary Table S2. Adjusted * incidence-rate ratios for acute exacerbation in 12 months if having singly increased FeNO levels (≥35ppb) compared with having normal FeNO levels and having singly increased blood eosinophil count (≥0.3 $\times$ 10^9/L) compared with having normal blood eosinophil count. Multivariable Poisson Regression Model was used.

		Acute exacerbation in 12 months	
FeNO (ppb)	Eosinophil count (/L)	Smoking status-adjusted	Multivariable adjusted
≥35		0.58 (0.45-0.75)	0.33 (0.23-0.48)
	≥0.3 $\times$ 10^9	0.88 (0.66-1.18)	0.75 (0.50-1.13)

Results in boldface indicate a P value less than 0.05.

* Multivariable adjustment included age, gender, BMI, smoking status, FEV_1 post-BD% pred, SGRQ, 6 minutes walking distance.

FeNO: exhaled nitric oxide fraction; BMI: body mass index; FEV_1: forced expiratory volume in 1 s; BD: bronchodilator; SGRQ: St. George's Respiratory questionnaire.

Supplementary Table S3. Adjusted * incidence-rate ratios for different levels of acute exacerbation in 12 months if having singly increased FeNO levels (≥35ppb) compared with having normal FeNO levels and having singly increased blood eosinophil count (≥0.3 × 10^9/L) compared with having normal blood eosinophil count. Multivariable Poisson Regression Model was used.

FeNO (ppb)	Eosinophil count (/L)	Acute exacerbation in 12 months					
		Smoking status- adjusted			Multivariable adjusted		
		Mild	Moderate	Severe	Mild	Moderate	Severe
≥35		0.33 (0.20-0.54)	0.33 (0.19-0.57)	1.08 (0.73-1.58)	0.25 (0.13-0.48)	-	0.61 (0.30-1.22)
	≥0.3 × 10^9	0.54 (0.31-0.94)	1.03 (0.58-1.82)	1.12 (0.74-1.71)	0.48 (0.24-0.99)	0.61 (0.30-1.24)	2.04 (0.90-4.61)

Results in boldface indicate a P value less than 0.05. * Multivariable adjustment included age, gender, BMI, smoking status, FEV_1 post-BD% pred, GRQ, 6 minutes walking distance. FeNO: exhaled nitric oxide fraction; BMI: body mass index; FEV_1: forced expiratory volume in 1 s; BD: bronchodilator; SGRQ: St. George's Respiratory questionnaire.

Supplementary Table S4. Adjusted * odds ratios (95% CI) for incidence of acute exacerbation in 12 months if having singly increased FeNO levels (≥ 35ppb) or blood eosinophil counts (≥ 0.3 × 10^9/L) or simultaneously increased FeNO levels and blood eosinophil counts compared with having both normal FeNO levels and blood eosinophil counts. Multivariable Logistic Regression Model was used.

	Incidence of exacerbation in 12 months	
	Smoking status- adjusted	Multivariable adjusted
FeNO<35ppb and Eosinophil count<0.3×10^9/L	1	1
FeNO ≥ 35 ppb or eosinophil count ≥ 0.3 × 10^9/L	0.50 (0.21-1.15)	0.39 (0.09-1.64)
FeNO ≥ 35 ppb and eosinophil count ≥ 0.3 × 10^9/L	0.08 (0.03-0.21)	0.03 (0.01-0.14)

Results in boldface indicate a P value less than 0.05.

* Multivariable adjustment included age, gender, BMI, smoking status, FEV_1 post-BD% pred, SGRQ, 6 minutes walking distance.

FeNO: exhaled nitric oxide fraction; BMI: body mass index; FEV_1: forced expiratory volume in 1 s; BD: bronchodilator; SGRQ: St. George's Respiratory questionnaire.

Supplementary Table S5. Adjusted * incidence-rate ratios (95% CI) for acute exacerbation in 12 months if having singly increased FeNO levels (/\\ 35ppb) or blood eosinophil counts (/\\ 0.3 × 10^9/L) or simultaneously increased FeNO levels and blood eosinophil counts compared with having both normal FeNO levels and blood eosinophil counts. Multivariable Poisson Regression Model was used.

	Acute exacerbation in 12 months	
	Smoking status- adjusted	Multivariable adjusted
FeNO<35ppb and Eosinophil count< 0.3×10^9/L	1	1
FeNO /\\ 35 ppb or eosinophil count /\\ 0.3 × 10^9/L	0.85 (0.64-1.13)	0.93 (0.64-1.35)
FeNO /\\35 ppb and eosinophil count /\\ 0.3 × 10^9/L	0.51 (0.35-0.76)	0.22 (0.12-0.40)

Results in boldface indicate a P value less than 0.05.

* Multivariable adjustment included age, gender, BMI, smoking status, FEV1 post-BD% pred, SGRQ, 6 minutes walking distance.

FeNO: exhaled nitric oxide fraction; BMI: body mass index; FEV1: forced expiratory volume in 1 s; BD: bronchodilator; SGRQ: St. George's Respiratory questionnaire.

Supplementary Table S6. Adjusted * incidence-rate ratios (95% CI) for acute exacerbation in 12 months if having singly increased FeNO levels (/\\ 35ppb) or blood eosinophil counts (/\\ 0.3 × 10^9/L) or simultaneously increased FeNO levels and blood eosinophil counts compared with having both normal FeNO levels and blood eosinophil counts. Multivariable Poisson Regression Model was used. table.

	Acute exacerbation in 12 months					
	Smoking status- adjusted			Multivariable adjusted		
	Mild	Moderate	Severe	Mild	Moderate	Severe
FeNO<35ppb and Eosinophil count< 0.3×10^9/L	1	1	1	1	1	1
FeNO /\\ 35 ppb or eosinophil count /\\ 0.3 × 10^9/L	0.65 (0.39-1.07)	0.75 (0.43-1.32)	1.10 (0.71-1.69)	0.52 (0.28-0.99)	1.12 (0.59-2.15)	1.35 (0.62-2.94)
FeNO /\\ 35 ppb and eosinophil count /\\ 0.3 × 10^9/L	0.20 (0.09-0.44)	0.31 (0.13-0.74)	1.17 (0.68-2.02)	0.11 (0.04-0.32)	0.04 (0.01-0.33)	0.96 (0.36-2.57)

Results in boldface indicate a P value less than 0.05.

* Multivariable adjustment included age, gender, BMI, smoking status, FEV_1 post-BD% pred, SGRQ, 6 minutes walking distance.

FeNO: exhaled nitric oxide fraction; BMI: body mass index; FEV_1: forced expiratory volume in 1 s; BD: bronchodilator; SGRQ: St. George's Respiratory questionnaire.

Supplementary Table S7. Predictive capabilities of increased F_{eNO} level and blood eosinophil count

	AUC (95% CI)	p-value versus F_{eNO}	p-value versus blood eosinophil count	p-value versus F_{eNO} and/or blood eosinophil count
F_{eNO}≥35 ppb	0.76 (0.69-0.83)	NA	0.99	0.17
Blood eosinophil count ≥0.3×10^9/L	0.76 (0.69-0.83)	0.99	NA	0.08
F_{eNO}≥35 ppb and/or Blood eosinophil count ≥0.3×10^9/L	0.79 (0.72-0.86)	0.17	0.08	NA

p-value was derived from testing the equality of two AUCs for the ROCs.

Logistic regression models were used to calculate the statistics and included smoking status as covariates.

F_{eNO}: exhaled nitric oxide fraction; AUC: area under the curve; ROC: receiver operating characteristic curve.

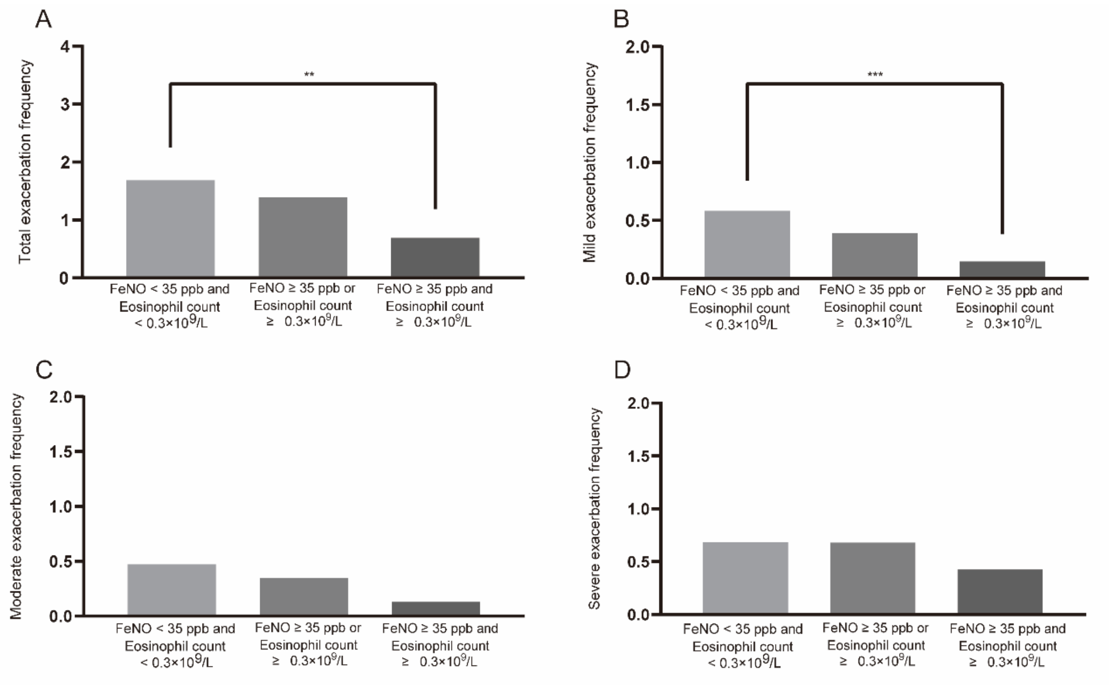

Supplementary Figure S1. Combination of normal or increased FeNO levels and blood eosinophil counts in relation to frequency of total acute exacerbation of COPD (**A**), frequency of mild acute exacerbation (**B**), frequency of moderate acute exacerbation (**C**) and frequency of severe acute exacerbation (**D**). ** $p < 0.01$ *** $p < 0.001$.

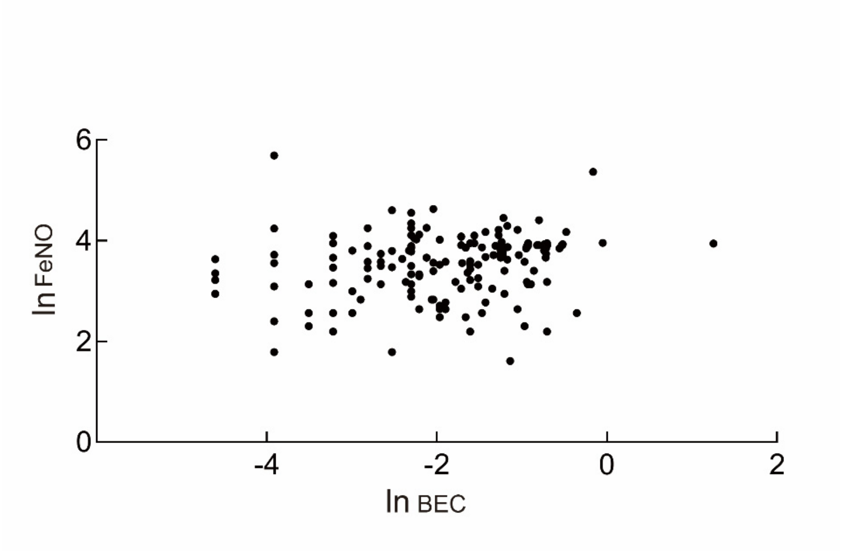

Supplementary Figure S2. Correlation between FeNO levels and blood eosinophil counts (r=0.35, p<0.001). FeNO: exhaled nitric oxide fraction; BEC: blood eosinophil count.

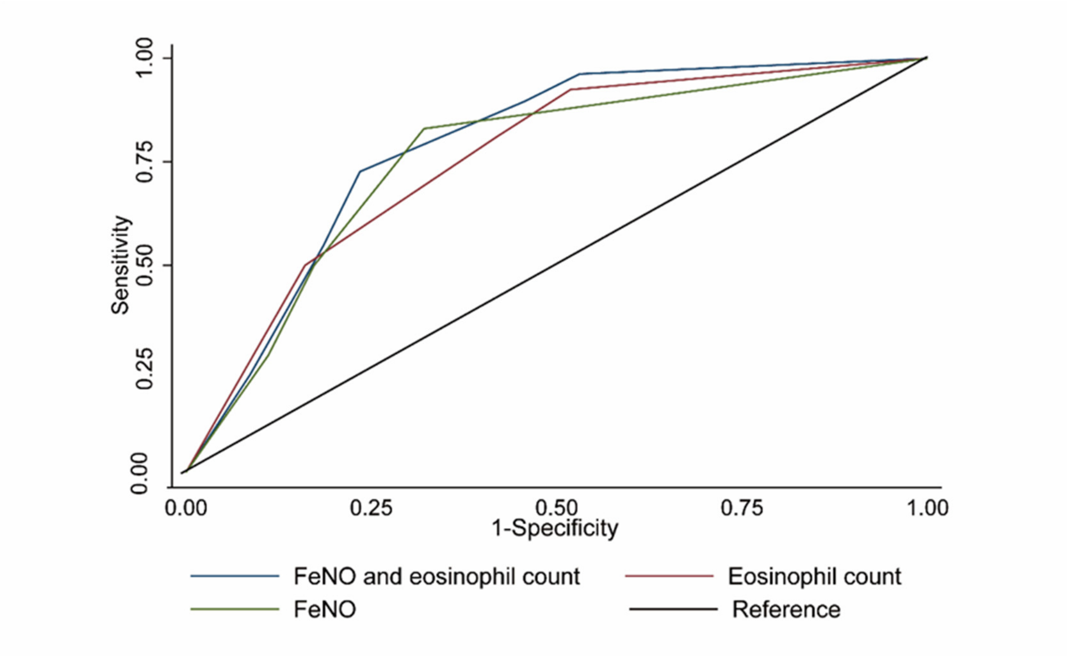

Supplementary Figure S3. Receiver operating characteristic curves (ROC) analysis for increased FeNO level and blood eosinophil count separately and combined. Logistic regression models were used to calculate the statistics and included smoking status as covariates. FeNO : exhaled nitric oxide fraction; AUC: area under the curve; ROC: receiver operating characteristic curve.
